# Axon demyelination and degeneration in a zebrafish *spastizin* model of hereditary spastic paraplegia

**DOI:** 10.1098/rsob.240100

**Published:** 2024-11-06

**Authors:** Vranda Garg, Selina André, Luisa Heyer, Gudrun Kracht, Torben Ruhwedel, Patricia Scholz, Till Ischebeck, Hauke B. Werner, Christian Dullin, Jacob Engelmann, Wiebke Möbius, Martin C. Göpfert, Roland Dosch, Bart R. H. Geurten

**Affiliations:** ^1^ Department of Cellular Neurobiology, Georg-August-University Göttingen, Göttingen, Germany; ^2^ Department of Developmental Biochemistry, Georg-August-University Göttingen, Göttingen, Germany; ^3^ Department of Neurogenetics, Max Planck Institute for Multidisciplinary Sciences, Göttingen, Germany; ^4^ Department of Plant Biochemistry, Georg-August-University Göttingen, Göttingen, Germany; ^5^ Institute of Plant Biology and Biotechnology, University of Münster, Münster, Germany; ^6^ Institute for Diagnostic and Interventional Radiology, University Medical Center, Göttingen, Germany; ^7^ Faculty of Biology, Bielefeld University, Bielefeld, Germany; ^8^ Institute for Humangenetics, University Medical Center, Göttingen, Germany; ^9^ Department of Zoology, University of Otago Dunedin, Dunedin, New Zealand

**Keywords:** Hereditary spastic paraplegia (HSP), spastizin, zebrafish, lower limb spasticity, mauthner neurons, C-start escape response

## Abstract

Hereditary spastic paraplegias (HSPs) are a diverse set of neurological disorders characterized by progressive spasticity and weakness in the lower limbs caused by damage to the axons of the corticospinal tract. More than 88 genetic mutations have been associated with HSP, yet the mechanisms underlying these disorders are not well understood. We replicated the pathophysiology of one form of HSP known as spastic paraplegia 15 (SPG15) in zebrafish. This disorder is caused in humans by mutations in the *ZFYVE26* gene, which codes for a protein called SPASTIZIN. We show that, in zebrafish, the significant reduction of Spastizin caused degeneration of large motor neurons. Motor neuron degeneration is associated with axon demyelination in the spinal cord and impaired locomotion in the *spastizin* mutants. Our findings reveal that the reduction in Spastizin compromises axonal integrity and affects the myelin sheath, ultimately recapitulating the pathophysiology of HSPs.

## 1. Introduction

Hereditary spastic paraplegias (HSPs) are a group of debilitating neurological disorders passed down through families. These conditions are clinically characterized by progressive weakness and spasticity in the lower limbs, making it difficult for affected individuals to move freely and perform daily tasks [1]. The cause of these symptoms is the distal degeneration of the longest corticospinal tract axons, which control lower limb motility [2–10]. To date, more than 88 different genes and 100 distinct spastic gait disease loci have been identified to cause HSP [11–16] in humans. The proteins encoded by these genes are involved in a variety of cellular processes [2,3,5,14].

A rare form of HSP is the so-called Kjellin syndrome or SPG15, which complicates the canonical symptoms of HSP with speech problems (dysarthria), mental impairments and pigmented maculopathy among others [17,18]. Experimental studies showed that the affected protein Spastizin is mostly involved in endosomal trafficking [19], autophagy [20,21], lysosomal biogenesis [22] and cytokinesis [23]. The 285 kDa protein is widely distributed inside cells and co-localizes with a variety of organelles including endosomes, microtubules, endoplasmic reticulum (ER), mid-body and vesicles involved in protein trafficking [17,24]. Spastizin interacts with Spatacsin and Beclin-1 [25,26] and is essential for the proper development and outgrowth of motor neuron axons in larval zebrafish [27].

To date, relatively few studies have addressed the pathophysiology of SPG15, with most of them using mouse models. For example, Khundadze *et al*. [19] created *zfyve26* knockout mice, that developed late-onset spastic paraplegia and cerebellar ataxia. Judging from this mutant, endolysosomal dysfunction might cause the degeneration of cortical motor neurons and Purkinje cells in the cerebellum. Despite considerable knowledge about the functions of Spastizin in different cellular processes, its role in the pathogenesis of HSP remains poorly understood.

In this study, we generated a model for the SPG15 in zebrafish and found that the significant reduction of Spastizin caused severe demyelination and degeneration of the spinal cord motor axons that leads to locomotion defects in *spastizin* mutants. Moreover, this truncating mutation affects the activity of the large motor neurons, including the M-cells, which are believed to be the functional reminiscent of lower limb motor neurons [28–30]. Our work characterizes the crucial role of Spastizin in maintaining neuronal integrity, emphasizing its profound impact on motor neuron function and underscoring its significance in the complex pathogenesis of HSP.

## 2. Material and methods

### 2.1. Zebrafish husbandry

The zebrafish were maintained according to the guidelines provided by the Westerfeld zebrafish book [31] and EuFishBioMed/Felasa [32], in compliance with the regulations of the Georg-August University, Goettingen and Bielefeld University, Bielefeld, Germany. Preliminary studies in our lab indicated a late onset of HSP-like symptoms in *spastizin* mutants, therefore, all the experiments were conducted on adult fish between 10 and 15 months of age.

### 2.2. *Spastizin* mutant generation

The target for *zfyve26* was 5’CTCACCGCGCTGCAGACA3’. *In vitro* transcribed guide RNA and Cas9 RNA were co-injected in +/−embryos produced by crossing homozygous males from another *spastizin* mutant line, known as *souffle^p96re^*, with wild-type AB*TLF females. The *souffle^p96re^* mutants carry a point mutation in the splice donor site, producing a shortened 2270 amino acid protein [33]. The injected embryos were developed to adulthood, and the success of the gene editing method was assessed using the T7 Endonuclease 1 (T7E1) assay kit (Invitrogen, Germany). Positive individuals from the resulting F1 generation were then crossed with wild-type fish to produce the F2 generation, which was screened again with T7E1 assay. The positive individuals in the F2 generation were crossed with each other to produce the F3 generation. In this generation, two males and four females were found to have a four-base pair deletion in one of the alleles, resulting in a premature stop codon. These heterozygous individuals were used for breeding and creating a new *spastizin* mutant line, which was named as *zfyve26^ge1^*.

### 2.3. Genotyping

The melting curve analysis method was used to identify heterozygous and homozygous *spastizin* mutants, using wild-type fish as a positive control (electronic supplementary material, figure S2). First, tail fin clips were cut and frozen at −80°C for 30 min. For extracting DNA, 100 µl lysis buffer containing 10 µl 0.1M Tris pH 8.5 (Carl Roth, Germany), 5 µl proteinase K (20 mg/ml) (Merck (Sigma-Aldrich), Germany), and 85 µl distilled water was added to them and the mixture was incubated at 55°C for 4 h. The proteinase K was inactivated by heating the mixture at 95°C for 10 min. A master mix containing 4.2 µl distilled water, 0.4 µM of each primer (Merck (Sigma-Aldrich), Germany) from a working concentration of 10 µM, 5 µl precision melt supermix (Bio-Rad, Germany) and 1 µl DNA was used for real-time PCR. The mixture was subjected to the following PCR conditions: 95°C for 3 min, 95°C for 5 s, 65°C for 10 s; repeat steps 2–3 39 more times; 95°C for 60 s; 65°C for 60 s in the presence of dsDNA intercalating dye SYBR green. Then a melt curve reaction was performed on the rehybridized DNA, by heating it from 65 to 95°C with 0.1°C increase every 5 s with plate read, and later analysed using Bio-rad CFX Maestro software. The following primer pair was used for the genotyping: Forward 5’-TCCAGGCGGGAGCTCTTC−3’ and Reverse 5’-ATCTTCTGCACGCGCCTC−3’.

### 2.4. RNA isolation, cDNA synthesis and quantitative PCR

To examine the expression levels of the *zfyve26* mRNA, RNA was isolated from the whole brain and spinal cord of one fish from each group (wild-type, heterozygous, homozygous) using the ZR Tissue & Insect RNA MicroPrep Kit (Zymo Research, USA). The tissues were transferred into a ZR BashingBead Lysis Tube with 800 µl RNA Lysis Buffer, homogenized in the TissueLyser LT (Qiagen, Germany) at 50 Hz for 2 min, and centrifuged for 2 min at 12 000 g. Approximately 600 µl of the supernatant was transferred to a Zymo-Spin IIIC Column in a collection tube and centrifuged for 1 min at 12 000 g. After discarding the column, 1 volume of 100% ethanol was added to the flow-through, mixed, and transferred to a Zymo-Spin IC Column, followed by centrifugation for 1 min at 12 000 g. The column was washed with 400 µl of RNA Wash Buffer, centrifuged for 1 min at 12 000 g, followed by a DNA digestion step with 40 µl of DNase I Reaction Mix (5 µl DNase I + 35 µl DNA Digestion Buffer), incubated at room temperature for 15 min.

Post-digestion, the column was washed with 400 µl of RNA Prep Buffer and centrifuged for 1 min at 12 000 g, followed by two washes with 700 and 400 µl of RNA Wash Buffer, respectively. The RNA was eluted with 15 µl of DNase/RNase-free water and centrifuged for 1 min at 10 000 g. mRNA concentration was measured with NanoDrop, showing higher concentrations in brain tissue than in spinal cord tissue.

The isolated mRNA was reverse transcribed into cDNA using the QuantiTect Reverse Transcription Kit (Qiagen, Germany). For brain tissue, 1 *µ*g of template RNA was used per reaction, and for spinal cord tissue, 250 pg per reaction. The genomic DNA elimination reaction mix was prepared, incubated at 42°C for 5 min, and set on ice. The reverse-transcription reaction was then performed at 42°C for 15 min and at 95°C for 3 min. The cDNA samples were stored at −80°C until quantitative PCR (qPCR) was performed.

Real-time qPCR was performed to detect and quantify target cDNA. Two primer pairs (zfyve26-1_FWD/REV and zfyve26-2_FWD/REV) were designed to amplify parts of the *zfyve26* gene of *Danio rerio* (electronic supplementary material, table S1). Housekeeping genes *α-tubulin*-1b and *elongation factor* 1*α* (EF1*α*) were used as controls. Primer efficiencies were tested, with zfyve26-2 and EF1 *α* showing 92% efficiency. The qPCR involved an initial denaturation at 95°C for 3 min, followed by 40 cycles of denaturation (10 s at 95°C), primer annealing (30 s at 55°C) and extension (30 s at 72°C). A melt curve analysis was performed starting at 55°C and increasing to 95°C to verify product specificity.

### 2.5. Western blot

Brain samples from all the three genotypes were homogenized in radio-immunoprecipitation assay (RIPA) buffer (0.1% sodium dodecyl sulfate (SDS) (Serva GmbH, Germany), 1% Nonidet P-40 (NP40) (Merck (Sigma-Aldrich), Germany), 1% sodium deoxycholate (Merck (Sigma-Aldrich), Germany), 150 mM sodium chloride (NaCl) (Carl Roth, Germany), 50 mM Tris (hydroxymethyl) aminomethane (THAM) hydrochloride (Tris-HCl) (pH = 7.2–7.5) (Carl Roth, Germany), Halt Protease Inhibitor Cocktail (100X) (Thermo Fisher Scientific, Germany)) and centrifuged at 10 000xg for 10 min at 4°C. The supernatant was collected and Bradford assay [34] was conducted to determine the protein concentration. 50 µg lysate from each sample type was separated on a 7% SDS-polyacrylamide gel initially at 30mA until the samples reached the separating gel and then at 40mA, a total of approximately 2 h. The samples on the gel were then electrotransferred to the polyvinylidene difluoride (PVDF) membrane (Bio-Rad, Germany) at 40V for 24 h in blotting buffer (5% MeOH, 0.05% SDS). For the overnight electrotransfer, the whole electrophoresis setup was placed in a cold room at 4°C, and the electrophoresis apparatus itself was placed an ice box. The membrane was blocked with 4% powdered milk (Carl Roth, Germany) in phosphate- buffered saline (PBS) (ChemSolute, Germany)+0.1% Tween (AppliChem, Germany) (PBST) for 30 min and incubated with anti-Spastizin (1 : 200, Invitrogen, Germany https://www.thermofisher.com/antibody/product/ZFYVE26-Antibody-Polyclonal/PA5-20685) antibody in 3% Bovine serum albumin (BSA) (Carl Roth, Germany) in PBST + 0.02% sodium azide (Carl Roth, Germany), overnight at 4°C. A secondary antibody, anti-rabbit horseradish peroxidase (HRP) (1 : 5000, Merck (Sigma-Aldrich), Germany), was used in the blocking solution for 30 min at room temperature. iBright CL1000 (Invitrogen, Germany) was used for capturing, and NIH ImageJ/FIJI [35] was used for analysing images.

A separate gel was used for immunoblotting with anti-*β*-tubulin (1 : 1000, DSHB, USA) antibody because while separating the bands with high molecular weight more clearly, the bands with low molecular weight were already leaked out. All the conditions and procedures were kept the same as for Spastizin except for the electrotransfer. Samples on the gel were electrotransferred to a nitrocellulose membrane for 1.5 h at 180mA in blotting buffer without additional methanol and SDS. Secondary antibody, anti-mouse horseradish peroxidase (HRP) (1 : 5000, Merck Sigma-Aldrich), Germany) was used. As the quantification was automated through FIJI, no bias mitigation methods were applied for data quantification.

### 2.6. Immunohistochemistry

The spinal cord was dissected and divided into four equal parts. The parts were fixed in 4% paraformaldehyde (PFA) (Carl Roth, Germany) in 0.1 M phosphate (PO_4_) buffer (pH = 7.4) and embedded in albumin-gelatin. 50 µm thick cross-sections of the spinal cord were cut using a Leica VT1000P vibrating blade microtome (Leica, Germany). Sections were blocked with 5% normal goat serum (NGS) (Jackson ImmunoResearch, UK) + 0.25% BSA in PBS/1% Triton X-100 (Merck (Sigma-Aldrich), Germany) for 2 h at room temperature. After blocking, the sections were incubated with anti-Spastizin primary antibody (1 : 300, Biogenes, Berlin, Germany [33]) in blocking solution and incubated at 4°C overnight on a stirrer. The following day, the sections were washed six times with PBS−1% Triton X-100 for 10 min each. The sections were then incubated with the secondary antibody donkey anti-rabbit AF 488 (1 : 300, Invitrogen, Germany) or donkey anti-rabbit Cyanine Dye 3 (Cy3) (1 : 300, Jackson ImmunoResearch, UK) in PBS/1% Triton X-100 in darkness for 3 h at room temperature. After incubation, the sections were washed six times with PBS for 10 min each and once with PBS/1,4-diazabicyclo[2.2.2]octane (DABCO) (Carl Roth, Germany) (1 : 1). Finally, the sections were mounted on slides with PBS/DABCO and stored in slide boxes at 4°C until the confocal imaging was performed. To study axons in the spinal cord, the same procedure was followed but using anti-*β*-Tubulin (1 : 1000, DSHB, USA) as the primary antibody and goat anti-mouse AF 546 (1 : 300, Invitrogen, Germany) as secondary antibody.

### 2.7. Confocal microscopy

Images were captured using a Leica TCS SP8 confocal microscope (Leica, Germany) at a resolution of 2048 × 2048 pixels with the 20× objective for Spastizin protein confirmation in the Mauthner axons. For axonal diameter measurement, images were acquired at a resolution of 1024 × 1024 pixels with the 63× glycerol immersion objective. NIH ImageJ/FIJI [35] was used for image processing and signal intensity measurement in the Mauthner axons. A MATLAB R2012b script (The MathWorks Inc., Natick, MA, USA) was used to measure axon diameter and numbers. The MATLAB script blinded the operator to the genotype of the spinal cord section.

### 2.8. Locomotion recordings

Zebrafish were filmed from above using a Genie HM1024 high-speed camera (Dalsa Imaging Solutions GmbH, Germany) linked with an Optem Zoom 125C 12.5:1 Micro-Inspection Lens System. The setups were illuminated from below with a light-emitting diode (LED) light plate (Lumitronix, Germany) and an aquarium light control (Elektronik-Werkstatt SSF, University of Göttingen, Germany). Videos were recorded for 30 s at 200 frames per second (fps). The experiments were conducted in the diurnal rhythm between 10.00 and 20.00.

#### 2.8.1. Unmotivated and motivated trials

To investigate the swimming behaviour, zebrafish were filmed for both cruising and motivated trials using the high-speed camera. The experiments were conducted in a 24.9 × 11.4 cm acrylic glass aquarium with a shallow water depth of 1.6 cm. The recording for cruising began 30 s after transferring a fish to the setup tank. The motivated trial started immediately after the cruising recording ended. For the motivated trials, a 474 g metal weight was used, that fell through a plastic tunnel and struck the setup table, creating a mechanical stimulus of 18.7 newtons (N) on the surface. By analysing the footage, the fish’s response to the mechanical stimulus was observed.

#### 2.8.2. Counter-current trials

A custom-built 17.2 × 4.4 cm Plexiglas aquarium complete with an installed water pump was constructed to study the impact of the water flow on fish movement. This setup allowed to simulation of three distinct current velocities of 172, 240 and 277 ml s^−1^, respectively, which were referred to as slow, medium and fast streams. The fish movement was recorded and analysed in these streams using a high-speed camera to understand how they adapt to different water flow conditions.

#### 2.8.3. Electrophysiology

The experimental set up for measuring the electric field potential from Mauthner neurons was adapted [54]. A set-up tank of 8 × 4 cm was used for the present study. The water jet from the picospritzer pressure pump (Parker Hannifin, USA) was used to evoke the escape response of the observed zebrafish. The signal was amplified 2000 times and band-pass filtered with a pass window of 300 to 500 Hz. Additionally, a Hum Bug (Quest Scientific, USA) was used to filter out the electronic noise of 50 Hz. The recording tank was filled with Milli-Q water to achieve a resistance of 18.2 MΩ cm^−3^. The animal movement was recorded at 936 fps for 5 s via the video camera that was triggered by the picospritzer. Electrophysiological signals were recorded with the micro2 1401 (Cambridge Electronic Design, UK) data acquisition (DAQ) system and analysed with Spike2 software (Cambridge Electronic Design, UK).

#### 2.8.4. Behavioural data analysis

Zebrafish locomotion was tracked with Limbless Animal traCkEr (LACE), a MATLAB R2012b script (The MathWorks Inc., Natick, Massachusetts, USA) [36]. As the quantification was fully automated through a consecutive software pipeline, no bias mitigation methods were applied for data quantification.

### 2.9. Extraction and analysis of cholesterol

Whole brain samples dissected from wild-type, heterozygous and homozygous *spastizin* mutant fish were kept in pre-weighed reaction cups and immediately flash-frozen in liquid nitrogen and stored at −80°C until further processing. The samples were lyophilized, weighed and then ground in a ball mill to make a fine powder. Two-phase extraction was performed by adding 400 µl of methyl-tert-butyl ether (MTBE): methanol (3 : 1, v/v; all solvents of High-performance liquid chromatography (HPLC)-grade (Thermo Fisher Scientific, Germany)), followed by vortexing and the addition of 200 µl water. 5 µg 17 : 0 free fatty acid (Merck, Germany) was used as the internal standard. After adding the standard, samples were mixed for 30 min on a rotator. The samples were then centrifuged at maximum speed for 1 min and the upper phase was transferred to a fresh tube and stored at −20°C until further processing. For gas chromatography-mass spectrometry (GC-MS) measurements, samples were prepared by evaporating 20−50 µl of the upper phase under a stream of nitrogen, redissolving in 10−15 µl anhydrous pyridine (Merck, Germany) and derivatizing with twice the volume of N-methyl-N-trimethylsilyltrifluoroacetamid (MSTFA) (Merck, Germany) to yield trimethylsilylated analytes. An Agilent 7890B gas chromatograph connected to an Agilent 5977N mass-selective detector was used for sample analysis, as described in [37]. Cholesterol was identified in comparison to an external standard. For quantification, the peak area of the mass-to-charge ratios 327 Da/e and 458 Da/e were used for the internal standard and cholesterol, respectively. Quantification was done using the Agilent software GC/MSD MassHunter with MSD ChemStation data analysis. Cholesterol values were normalized to the internal standard and the mass of the sample. As the data quantification was automated, no bias mitigation methods were applied for data quantification.

### 2.10. High-pressure freezing and electron microscopy

After sacrificing zebrafish, the spinal cord was dissected into three segments using the number of vertebrae for orientation. A 3 mm long piece of the proximal and distal part of the spinal cord was high-pressure frozen with the Leica HPM100 (Leica, Germany) using 20% polyvinylpyrrolidone (PVP; M_W_ 10,000) (Sigma-Aldrich, Germany) in PBS as a cryoprotectant. The samples were then freeze-substituted using the Leica AFS2 (Leica, Germany) and embedded in Epon (Serva GmbH, Germany), as previously described [38]. Semithin (0.5 µm) or ultrathin (50 nm) sections of Epon-embedded tissue were cut using the Leica UC7 ultramicrotome (Leica, Germany) and contrasted with UranylLess stain (Science Services GmbH, Germany). Electron micrographs were acquired using the electron microscope LEO EM912AB (Carl Zeiss Microscopy GmbH, Germany) equipped with a wide-angle dual speed 2k-charged-coupled device (CCD)-camera (TRS, Germany). No bias mitigation methods were applied for data quantification.

### 2.11. Micro-computed tomography scan

Zebrafish were sacrificed, briefly rinsed in water and then transferred to 35 and 70% ethanol for 1 h each. Staining and fixation were carried out by placing fish for approximately 10 days at room temperature (RT) under slow rotation in a 4% PFA solution (Serva Electrophoresis, Germany) in PBS, pH 7.4 (Invitrogen, Germany), containing 0.7% phosphotungstic acid (PTA) solution (Merck (Sigma-Aldrich), Germany) diluted in 70% ethanol. Afterwards, fish were briefly rinsed in water and embedded in 1% agarose (Carl Roth, Germany) in 1.8 ml vials (Nunc CryoTube Vials, Merck (Sigma-Aldrich), Germany). For scanning, the *in vivo* micro-omputed tomography (CT) system Quantum FX (Perkin Elmer, USA) was used with the following settings: tube voltage 90 kV, tube current 200 µA, field of view (FOV) 10 × 10 mm^2^, total acquisition time 3 min, resulting in a reconstructed pixel size of 20 µm and an image matrix of 512 × 512 × 512 voxel [39]. A custom-made Python script was used to stitch 2–3 consecutive scans together. This script, in addition to finding the perfect overlap also corrects for drift in between the scans. Data were analysed using NIH ImageJ/FIJI software [35].

### 2.12. Statistics

Statistical significance for all data sets presented in this study was calculated using Fisher’s permutation test [40–42] except for mass spectrometry analysis of cholesterol, which was calculated using Wilcoxon rank-sum [43] and the Western Blot quantification which was analysed with an ANOVA followed by Tukey’s honestly significant difference (HSD) test for post hoc comparisons. For the permutation test, the original samples were bootstrapped 2 00 000 times. All *p*-values were corrected with the Benjamini–Hochberg false discovery rate correction [44].

## 3. Results

### 3.1. Loss of spastizin from the brain and spinal cord of *spastizin* mutants

**Figure 1.**
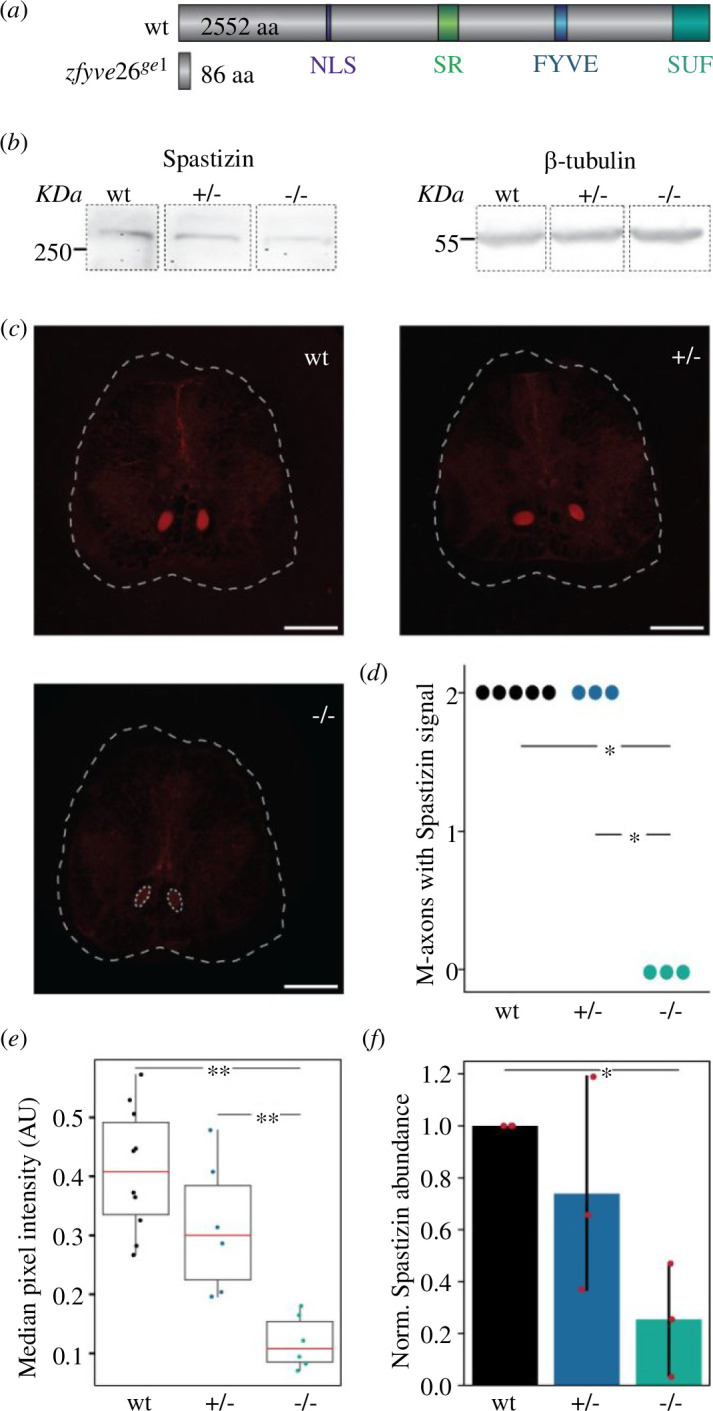
Effects of CRISPR-Cas9 mediated gene editing on Spastizin abundance. (*a*) A four-basepair deletion in the second exon of *zfyve26* leads to a frameshift mutation that introduces a premature stop codon after 86 amino acids. (*b*) Immunoblotting of brain tissue, showing reduced levels of Spastizin protein in the homozygous mutants. *β*-tubulin is used as the loading control. Complete gel is shown in electronic supplementary material, figure S4. (*c*) Representative images of the immunohistochemical analysis of the sections of the spinal cord using anti-Spastizin primary antibody and donkey anti-rabbit Cyanine Dye 3 (Cy3) secondary antibody. The white dashed lines mark the section of the spinal cord. The red area illustrates the Mauthner axons with Spastizin abundance. Although Spastizin was abundant in both the axons in wild-type and heterozygous mutant fish, its abundance was significantly reduced in the axons in homozygous mutants, marked by the white-dashed circles (scale bar: 100 µm). (*d*) Quantification for the number of Mauthner axons having a signal for Spastizin in each genotype. The dots represent the number of animals examined from each genotype. (*e*) Box plot of the median pixel intensity of the Spastizin signal in the two M-cell axons, normalized to the maximum intensity. The red line represents the median of all values, the box displays the upper and lower quartile, the whiskers denote 1.5 times the interquartile range and the dots show individual data points. There is a significant reduction of the signal intensity for Spastizin in the M-cell axons of homozygous mutants compared to both heterozygous and wild-type fish (no. of fish: wt = 5, +/− = 3, −/− = 3). (*f*) The bar graph shows the relative abundance of Spastizin protein, normalized to anti-*β*-tubulin, between the three genotypes. Data are depicted as mean and variance, with red dots representing the individual data points. There is a significant decrease in the abundance of 1S2pastizin in the brain of mutant fish compared to the wild-type (no. of biological replicates = 3). Statistical significance was tested with an ANOVA followed by Tukey’s HSD for post hoc comparisons of Spastizin abundance and by Fisher’s permutation test for the number of M-cell axons with Spastizin signal and its intensity in the M-cell axons. *p < 0.05, **p < 0.01.

In this study, we sought to uncover the role of SPASTIZIN in the pathogenesis of HSP, a debilitating disorder in humans characterized by progressive lower limb spasticity and weakness. We previously established that *souffle* is the zebrafish orthologue of the HSP-associated gene, *ZFYVE26,* which encodes SPASTIZIN [33]. Further analysis of the p96re allele of *souffle*/*suf* revealed that Spastizin lost only 282 of 2552 amino acids in the full-length protein and no significant differences in the locomotion of *suf* mutants were found when compared to the wild-types. We, therefore, generated a stronger mutant allele named *zfyve26^ge1^*. Molecular characterization of the mutated *zfyve26* gene revealed a four nucleotide deletion in the second exon, leading to a frameshift mutation that introduced a premature stop codon after amino acid 86 (figure 1*a*; electronic supplementary material, figure S1). We confirmed the deletion via Sanger sequencing (electronic supplementary material, figure S1D). Together, these results suggest that *zfyve26^ge1^* is a mutant allele with reduced Spastizin activity. To test this hypothesis, we conducted an exploratory quantitative PCR (qPCR) study, which suggested elevated levels of mRNA in the brain and spinal cord of the mutants (electronic supplementary material, figure S3). For knockout zebrafish mutants, an increase in mRNA levels has been reported before [45], indicating cellular mechanisms that compensate for the lack of protein in cells [46]. Indeed, we found a significant decrease in the abundance of Spastizin protein in the brain and spinal cord of the mutants, analysed using Western blot and immunohistochemistry, respectively. Whereas in Western blots a band of the expected size (>250 KDa) was visible in both wild-type and heterozygous mutant extracts, the bands intensity was significantly reduced for homozygous mutants (figure 1*b*,*f*; complete gel in electronic supplementary material, figure S4), indicating that our mutation is not a complete knockout. As a loading control, we used anti-*β*-Tubulin protein, for which a band of 55 KDa was observed in the brain tissue of each genotype (figure 1*b*). To analyse the spatial abundance of Spastizin, we examined sections of spinal cords with anti-Spastizin antibodies. Remarkably, we observed high abundance of Spastizin protein in the large axons of the M-cells of wild-type fish (figure 1*c,d*; electronic supplementary material, figure S5). Moreover, Spastizin abundance was significantly reduced in the M-cell axons of the homozygous mutants, although the structure of the M-axons is still visible in the spinal cord section. Quantitative analysis of the staining revealed a statistically significant reduction of Spastizin in homozygous mutants, further supporting the notion that Spastizin is not present in the neuronal tissue (figure 1*e*). Taken together, our results suggest that Spastizin protein is highly abundant in M-cells and this abundance is lost in *zfyve26^ge1^* mutants.

### 3.2. Locomotion defects in *spastizin* mutants

**Figure 2.**
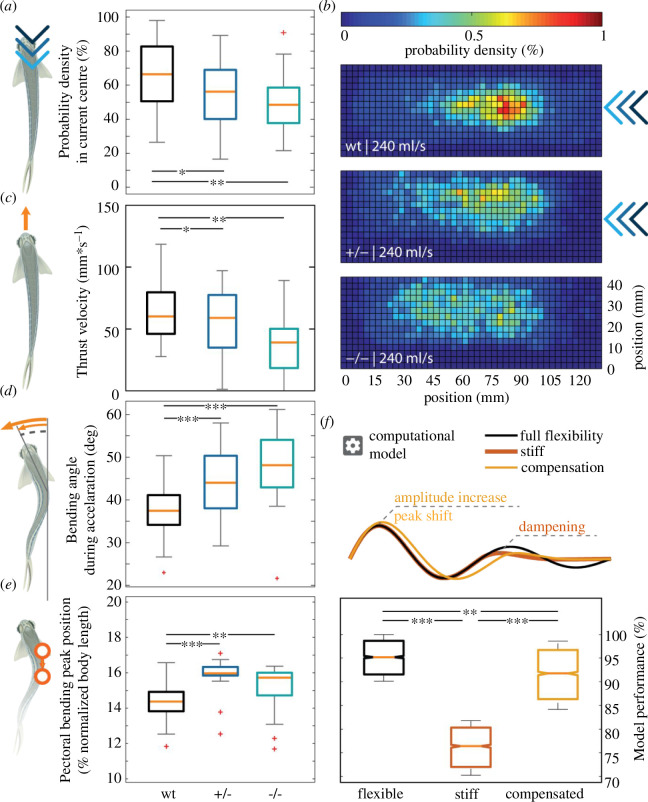
Locomotion defects observed in *spastizin* mutants during unmotivated and counter-current trials. Data are represented as box plots for assessing various aspects of locomotive ability of wild-type and *spastizin* mutants. The orange line shows the median of all individuals, the box displays the upper and lower quartile, the whiskers denote 1.5 times the interquartile range, the plus-sign marks outlier. (*a*) The homozygous mutants spend less time in the centre of the water current as compared to heterozygous and wild-type fish. (*b*) Each histogram is normalized to 100% and the colour bar goes from 0 to1%. The heat map shows all possible locations of the fish in the setup. Blue colour indicates low probability and red colour illustrates high probability of presence. Whereas the *spastizin* mutants are more dispersed within the setup, the wild-type fish stay more at the centre, where the current is most constant. (*c*) Decrease in the median thrust velocity of homozygous *spastizin* mutants as compared to heterozygous and wild-type fish during unmotivated trials. (*d*) Increase in the bending angle for both heterozygous and homozygous mutants compared to wild-types. (*e*) Increase in pectoral bending peak position (normalized to body length) of heterozygous and homozygous mutants compared to wild-types. (*f*) Computational model depicting a shift in the peak position of pectoral standing wave, which compensates for the dampened travelling wave. The box plot shows the model performance with fully flexible and compensated at the same level and high as compared to the stiff (no. of fish: wt = 53, +/− = 23, −/− = 21). Statistical significance was tested with Fisher’s permutation test. *p < 0 .05, **p < 0.01, ***p < 0.001.

To address whether Spastizin is required for locomotion in zebrafish, we first tested the ability of the animals to swim against a water stream that induced their natural rheotaxic behaviour [47]. In *spastizin* mutants, we observed a significantly reduced ability to remain in the centre of the stream (figure 2*a*). Using two-dimensional (2D) heat maps of the probability density for every possible position along the setup, we found that while wild-type zebrafish preferred to remain in the centre of the current, heterozygous and homozygous mutants avoided it (figure 2*b*). Zebrafish create translational thrust through undulatory sinus-like waves of their body. This motion is composed of a standing and a travelling wave [48–53]. The forward velocity in zebrafish mutants is only slightly decreased compared to wildtype fish (figure 2*c*) and was not observed.

in juvenile fish up to the age of 44 weeks (electronic supplementary material, figure S6). Our analysis further revealed that the mutants bend their body more strongly to achieve the same acceleration as the wild-type fish (figure 2*d*), suggesting that the increased pectoral standing wave might be compensating for an otherwise caused loss in locomotion ability. To substantiate this interpretation, we developed a computational model of the propulsion with either a reduced travelling wave (stiffness) or with the reduced travelling wave being supplemented by an increased standing wave (pectoral fin). This confirmed that a standing wave of the same angle but with a higher amplitude could compensate for the loss of the travelling wave in the mutants (figure 2*f*, compensatory wave). For this, the peak of the standing wave would have to travel more caudally (figure 2*f*). Indeed, the data of heterozygous and homozygous mutants showed that the pectoral bending peak shifts more caudally compared to the wild-types (figure 2*e*).

### 3.3. Functioning of M-cells affected in *spastizin* mutants

M-cells drive the C-start escape response [28,29]. Because these cells are giant neurons, their field potential can be measured in freely swimming fish using the electrodes attached to the fish tank [54]. Here, we exploit this to investigate if the lack of Spastizin from the M-cells resulted in altered escape behaviour and/or could be linked to altered M-cell activity in evoked escape responses. Escape behaviour in zebrafish was elicited by administering an air puff into the tank using a micro spritzer pump.

**Figure 3.**
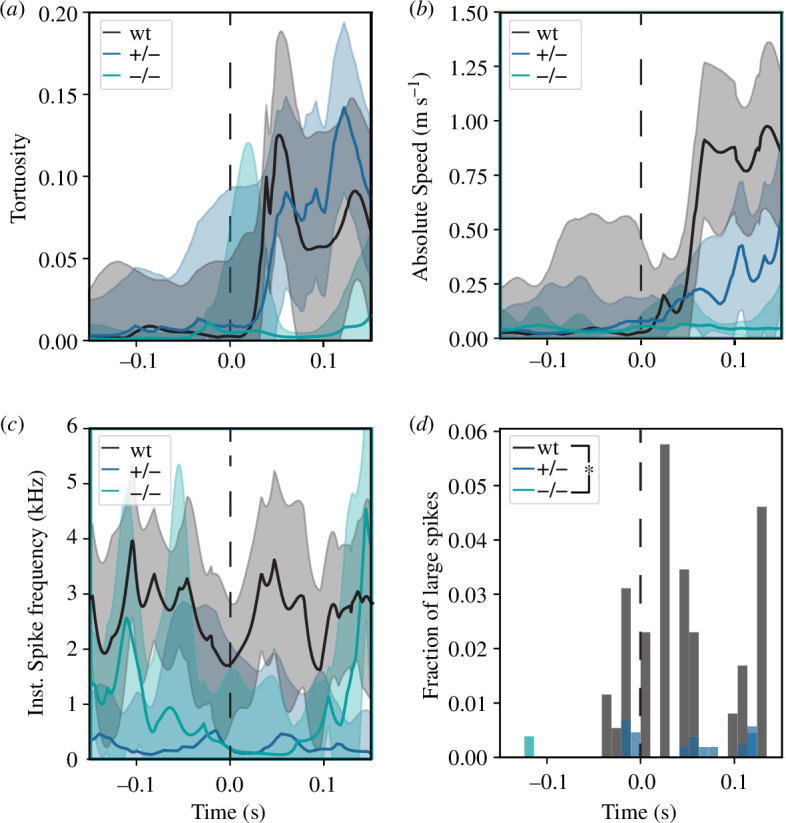
Lack of Spastizin affects the function of M-cells. (*a*) shows the tortuosity of the zebrafish over time as median with the surrounding 95% confidence interval. Tortuosity is defined as the difference in the body length and the distance between the head and tail of the animal, normalized to the body length. A value of 0 signifies the animal is entirely straight and 1 tortuosity signifies that head and tail touch. (*b*) presents the absolute velocity during the same time window. (*c*) shows the instantaneous spike frequency over time, as in (*a*) The dashed vertical line marks the time of the stimulus presentation. (*d*) shows the mean histogram of the occurrence of large spikes during the same time window. All three parameters are low for homozygous mutants compared to heterozygous and wild-types (no. of fish: wt = 9, +/− = 18, −/− = 5). Statistical significance was tested with Fisher’s permutation test (*p < 0.05) and showed a significant reduction in large spikes in mutants compared to wild-type animals.

Contrary to wild-type fish, the mutants could not form a C-bend in response to the stimulus, as can be seen by the tortuosity of their silhouette, measured as (head to tail distance − body length) ÷ body length (figure 3*a*). Since the C-bend is crucial for the rapid acceleration during the escape response, mutants exhibit a discernibly slower movement compared to the wild-type (figure 3*b*). In addition, we find that while both the small and large spike frequencies were lower in spastizin mutants (figure 3*c,d*; elecronic supplementary material, figure S7), only large spikes occurred coupled to the stimulus (time 0). These observations document that Spastizin influences on the activity of the M-cells, with its significant reduction contributing to a distinctly slower escape response.

### 3.4. Degeneration of lower motor neuron axons and their myelin sheaths in the *spastizin* mutants

**Figure 4.**
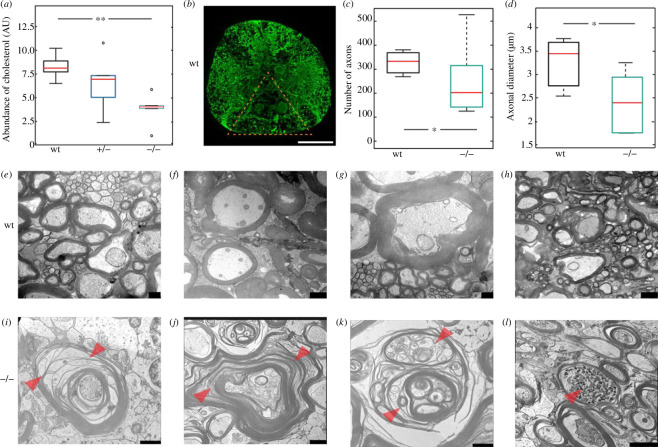
Degeneration and demyelination of the spinal cord motor neuron axons of the *spastizin* mutants. (*a*) Data are represented as box plots for the abundance of cholesterol in the brain of wild-type and *spastizin* mutant fish, divided by the mass of the sample and normalized to the internal standard. The red line depicts the median, the box displays the upper and lower quartile and the whiskers denote 1.5 times the inter quartile distance. Black circle marks outlier. There is a significant reduction in the abundance of cholesterol in the brain of homozygous mutants compared to the wild-types (no. of fish: wt = 5, +/− = 5, −/− = 5). (*b*) Representative image of immunohistochemical analysis of spinal cord cross sections stained with a neuronal marker anti-*β*-tubulin. The triangle marks the area in which the number and diameter of axons were quantified (scale bar: 100 µm). (*c,d*) Data are represented as box plots for number and diameter of axons in the evenly distributed sections of the spinal cord for wild-type and mutant zebrafish. The red line depicts the median, the box displays the upper and lower quartile and the whiskers denote 1.5 times the inter quartile distance. There is a significant decrease in the number and diameter of axons in the *spastizin* mutants compared to the wild-types (no. of fish: wt = 3, −/− = 6). (*e–l*) Representative images of the cross-section of the spinal cord showing large-calibre axons surrounded with myelin sheath (no. of fish: wt = 1, −/− = 2). (*e,f,g,h*) Representative images of intact myelin sheath in the wild-type fish. (*I,g*) Arrowheads mark fragmentation or splitting of myelin sheath in the homozygous mutants. (*k*) Severe vesiculation profile of myelin sheath marked by arrowheads, in homozygous mutants. (*l*) Arrowhead indicates swelling in large-calibre axons of the spinal cord of homozygous *spastizin* mutants. Scale bars: 250 nm in (*e,g*), 1 µm in (*f,h,i,k*) and 2 µm in (*j,l*). Statistical significance was tested with Wilcoxon rank-sum test for cholesterol abundance and with Fisher’s permutation test for the number and diameter of axons *p < 0.05 and **p < 0.01.

Axon degeneration of the corticospinal motor neurons in a length-dependent manner is the prime cause of spasticity and locomotion defects in HSP patients [2,3,6,9,10]. Interestingly, we also found a significant decrease in the number and diameter of axons in the spinal cord of the *spastizin* mutants (figure 4*a–c*), confirming axonopathy in the zebrafish model of SPG15. We also found a significant decrease in the abundance of cholesterol in the brain of *spastizin* mutants (figure 4*d*), and we thus investigated the cellular structure of the neurons’ axons in the spinal cord. Compared to the intact myelin observed in wild-types (figure 4*e–h*) axons, severe splitting and vesiculation, two hallmarks of demyelination, were present in the large-calibre axons of mutant spinal cord motor neurons (figure 4*i–k*). In addition to demyelination, we found axonal swelling in the *spastizin* mutants (figure 4*l*), a further distinctive feature of neuropathology. Our results thus indicate that Spastizin is essential in the maintenance of the myelin sheath and axonal integrity. Together, these results suggest that the locomotion defects are caused by demyelination and degeneration of the lower motor neurons’ axons in the *spastizin* mutants.

### 3.5. No skeletal deformities in *spastizin* mutants

To further validate that the locomotion defects in *spastizin* mutants are caused by neuronal defects rather than skeletal abnormalities, we utilized micro-CT scanning to analyse the vertebral column of wild-type and *spastizin* mutants. Our analysis revealed no distinguishable differences in the skeletal structure between wild-type and heterozygous or homozygous mutants (electronic supplementary material, figure S8). In addition, we examined the muscle area in the caudal region of the fish. It was unaltered in the mutant fish (electronic supplementary material, figure S9), indicating absence of muscular atrophy. Hence, instead of reporting muscle defects, the observed behavioural phenotypes seem to be caused by neuronal defects.

## 4. Discussion

HSP is a complex disorder associated with more than 88 genes and several cellular pathways [2,3,5,11–16]. In this study, we focused on a rare form of HSP called SPG15. To date, three SPG15 models have been reported: one in mice [19] and another two in zebrafish [27,33]. Both our zebrafish model and the mouse model exhibit late-onset ataxia. The mouse model shows a reduction in large-diameter axons in the lumbar corticospinal tract, similar to the reduction observed in our zebrafish spinal cord sections. While they used transmission electron microscopy (TEM) to show degenerating spinal cord axons [19], we used high-pressure freezing and electron microscopy (HPF-EM), which preserves myelin, revealing demyelination, axon swelling and degeneration in large-calibre spinal cord axons. Martin *et al*. [27] reported a zebrafish model generated using morpholino injection, which is now considered less reliable due to off-target effects [27]. In contrast, we developed the first CRISPR-Cas9-mediated stable SPG15 model. The morpholino larvae showed neurodevelopmental phenotypes such as motor neuron outgrowth defects and neuromuscular junction issues [27], which we did not find evidence for. Our model, however, exhibits late-onset stiffness, demyelination, axon degeneration, and reduced cholesterol, indicating different underlying mechanisms. During a maternal mutant screen, Dosch *et al*. [55] found an oocyte maturation defect, which was further analysed by Kanagaraj *et al*. [33]. Their study did not find a locomotion phenotype in the larvae, which is consistent with our findings that the locomotion defect occurs at a late stage and is masked by compensatory movements. Kanagaraj *et al*. [33] focused their study on the oocyte and larval stages, thereby augmenting our findings and providing a more complete picture of the pathophysiology of SPG15 mutant models in zebrafish.

The respective pathophysiological mechanisms, however, are little understood, hampering therapeutic treatments of HSP [56,57]. Using zebrafish as a model system, we found that Spastizin protein is particularly abundant in the large-calibre axons of the Mauthner-cells, and that its reduction is associated with degeneration of the myelin sheath and locomotion defects, recapitulating key disease features of HSP. Studies in zebrafish oocytes, mouse models, and patient-derived cells have suggested that Spastizin plays a crucial role in various cellular processes, such as membrane trafficking [19,33], autophagosome maturation [20,21], lysosomal regeneration [22], cytokinesis [23], which all seem particularly important for the survival and maintenance of large neurons [58–60]. We speculate that Spastizin mutation could cause demyelination and cell degeneration through one of these mechanisms. Future studies can now utilize Mauthner cells [30] to determine which of the cellular processes affected by Spastizin are the ultimate and proximate causes of the observed phenotypes.

Apparently, *spastizin* mutants have less endurance than wild-types in constantly swimming against the water stream (see figure 2*a*). Zebrafish generate translational thrust by producing a standing wave and a travelling wave [48–53]. Wild-type zebrafish create both waves during movement (see figure 2*f*, full flexibility wave). We hypothesize that the travelling wave is dampened at the caudal end of *spastizin* mutants (see figure 2*f*, stiff wave), which would be consistent with the longest motor neurons degenerating first. While a complete loss of the abdominal travelling wave would lead to a severe reduction of thrust speed, we observed only a moderate reduction in thrust (see figure 2*c*). Possibly, the mutant fish compensate for their caudal stiffness with a larger pectoral wave. This is further corroborated by our simple mathematical model, which predicts a shift in the apex of the caudal wave due to an increased amplitude, as observed in the mutant (see figure 2*e*). This compensatory mechanism potentially reduces propulsion efficiency and prevents mutants from reaching the same rheotactic endurance as wild-type fish (see figure 2*a*).

The observed dysfunction in Mauthner cells parallels the loss of limb movement control in human patients [2–10]. Frogs, uniquely possessing both Mauthner cells and legs, provide a critical model for our understanding: Post-metamorphosis, these cells in frogs are known to control limb movements [61,62], thereby bolstering the argument that Mauthner cells offer a viable model for studying lower limb control issues characteristic of HSP. The study also noted an impact on other large motor neurons, contributing to reduced mobility during normal cruising behaviour. This reduction, however, is often masked by compensatory movements in the fish, potentially explaining the limited research on adult fish in the context of motor neuron degeneration. Despite such compensation mechanisms, a notable decrease in thrust velocity was observed in the mutants. To ascertain that this decrease stems from motor neuron dysfunction rather than skeletal issues, CT scans were conducted. We found no significant differences in the vertebral column or muscle mass between wild-type and *spastizin* mutants, confirming that the locomotion defects arise from motor neuron degeneration rather than skeletal deformities.

Recent studies implicate altered lipid metabolism, particularly cholesterol, in HSP pathophysiology [63–67]. Cholesterol, vital for membrane and myelin formation [68–71], is affected in *spastizin* mutants, with reduced brain cholesterol levels hinting at disrupted regulatory mechanisms. This aligns with Spastizin’s localization at the ER, potentially influencing cholesterol metabolism [17,24]. Furthermore, our findings of demyelination and neuropathology in *spastizin* mutants spinal cords hint at a novel role for Spastizin in myelin integrity, potentially linked to cholesterol abundance. The interplay of Spastizin with calcium homeostasis, akin to its interaction partner Spatacsin’s effect on calcium and cholesterol levels [72], warrants further investigation to elucidate its role in HSP pathogenesis.

## 5. Conclusions

Overall, our study provides a valuable new model to study SPG15 in adult zebrafish at behavioural, neuronal and biochemical levels. Our study suggests that depreciation of Spastizin from the large-calibre axons is associated with a disturbance in the myelin abundance, which affects the neuronal integrity, and coincides with the degeneration of large-calibre motor neuron axons. The combined effects of these disturbances lead to locomotion problems. Our study opens novel avenues to address the cellular and biochemical processes affected by HSP and establishes a novel tool to develop therapeutic strategies for the benefit of HSP patients.

## Data Availability

Raw data will be provided on request to the corresponding author. Data are also provided in Figshare [73]. Supplementary material is available online [74].
